# Co-targeting WIP1 and PARP induces synthetic lethality in hepatocellular carcinoma

**DOI:** 10.1186/s12964-022-00850-2

**Published:** 2022-03-28

**Authors:** Miaoqin Chen, Weikai Wang, Shiman Hu, Yifan Tong, Yiling Li, Qi Wei, Lei Yu, Liyuan Zhu, Yiran Zhu, Leiming Liu, Zhenyu Ju, Xian Wang, Hongchuan Jin, Lifeng Feng

**Affiliations:** 1 Laboratory of Cancer Biology, Key Lab of Biotherapy in Zhejiang Province, Cancer Institute of Zhejiang University, Sir Run Run Shaw Hospital, Cancer Center, School of Medicine, Zhejiang University, Hangzhou, 310016 Zhejiang China; 2 Department of Medical Oncology, Sir Run Run Shaw Hospital, Medical School of Zhejiang University, Hangzhou, China; 3 Department of General Surgery, Sir Run Run Shaw Hospital, Medical School of Zhejiang University, Hangzhou, China; 4 Institute of Aging and Regenerative Medicine, Jinan University, Guangzhou, China

**Keywords:** WIP1, DNA damage repair, PAPR inhibitor, Hepatocellular carcinoma

## Abstract

**Background:**

Hepatocellular carcinoma (HCC) is one of the most fatal cancers. Due to limited strategies for effective treatments, patients with advanced HCC have a very poor prognosis. This study aims to identify new insights in HCC to develop novel strategies for HCC management.

**Methods:**

The role of WIP1 (wild type p53 induced protein phosphatase1) in HCC was analyzed in HCC cells, xenograft model, DEN (Diethylnitrosamine) induced mice liver cancer model with WIP1 knockout mice, and TCGA database. DNA damage was evaluated by Gene Set Enrichment Analysis, western blotting, comet assay, and Immunofluorescence.

**Results:**

High expression of WIP1 is associated with the poor prognosis of patients with HCC. Genetically and chemically suppression of WIP1 drastically reduced HCC cell proliferation. Besides, WIP1 knockout retarded DEN induced mice hepato-carcinogenesis. Mechanically, WIP1 inhibition induced DNA damage by increasing H2AX phosphorylation (γH2AX). Therefore, suppression of WIP1 and PARP induced synthetic lethality in HCC in vitro and in vivo by augmenting DNA damage.

**Conclusion:**

WIP1 plays an oncogenic effect in HCC development, and targeting WIP1-dependent DNA damage repair alone or in combination with PARP inhibition might be a reasonable strategy for HCC management.

**Video abstract**

**Supplementary Information:**

The online version contains supplementary material available at 10.1186/s12964-022-00850-2.

## Background

Liver cancer is one of the most common malignance worldwide [1]. Hepatocellular carcinoma (HCC) accounts for 85%-90% of liver cancer. HCC is an aggressive cancer associated with poor survival, frequent recurrence, and high incidence of metastases after surgical resection or chemotherapy [2]. Hence, understanding the molecular mechanisms of HCC pathogenesis is urgently needed to develop novel clinical strategies.

Wild type p53 induced protein phosphatase 1 (WIP1), also known as protein phosphatase magnesium-dependent 1δ (PPM1D), is a member of the PP2C family of Ser/Thr protein phosphatases [3]. It was found to dephosphorylate many proteins and thus implicated in various physio-pathology processes such as DNA damage, immunity, autophagy and so on [4–6]. The expression of WIP1 can be induced by a variety of stresses through p53, p38 MAPK [7], c-Jun [8] and NF-κB [9] pathways. For example, the expression of WIP1 could be up-regulated in a p53-dependent manner in response to ionizing radiation [3]. Interestingly, WIP1 overexpression would inactivate p53 to promote tumorigenesis via abrogating p53-dependent apoptosis and cell-cycle arrest. In addition, it can directly dephosphorylate many other proteins critical for cancer development, such as p53 [10], mTOR [11, 12], H2AX [13], p38 MAPK [14, 15], chk1 [16], chk2 [17], and UNG2 [18]. Moreover, *PPM1D* amplification is found in several solid tumors, including medulloblastoma [19], neuroblastoma [20], pancreatic adenocarcinoma [21], ovarian clear cell carcinoma [22] and breast cancer [11, 23]. WIP1 is up-regulated in HCC, and high expression of WIP1 was associated with a more advanced tumor-node-metastasis stage and poor prognosis [24–27]. Wang et al. reported that down-regulated microRNA-29c up-regulates its target gene *PPM1D* expression in HCC, and overexpression of microRNA-29c could decrease WIP1 expression and inhibit HCC cell proliferation [24]. However, the mechanism of WIP1 in HCC development is unclear.

In the current study, we identified that WIP1 depletion inhibited HCC development via increasing DNA damage by suppressing γH2AX dephosphorylation. In addition, WIP1 inhibition in combination with PARP inhibitors induces HCC synergy lethal in vitro and in vivo. Therefore, targeting WIP1 dependent DNA damage repair might be a novel strategy for the clinical management of HCC.

## Methods

### Cells, antibodies, plasmids and chemicals

Human liver cancer cell lines including PLC/PRF/5, QGY7703, Huh7, etc., normal liver cell line Chang and mouse liver cancer cell line Hepa1-6 were all purchased from Cell Bank of the Typical Culture Preservation Committee, Chinese Academy of Sciences (Shanghai, China). PLC/PRF/5, Huh7,Hepa1-6, HepG2, SK-Hep1 and HCCLM3 cells were cultured in the DMEM medium (Invitrogen, Shanghai, China).And Chang, QGY7703, BEL7402 were cultured in RPMI1640 medium (Invitrogen, Shanghai, China). All mediums were supplemented with 10% FBS and 100U/mL penicillin–streptomycin. The following antibodies were used for Western blotting: WIP1 (sc-376257) to detect human samples from Santa Cruz (Shanghai, China); WIP1 (A6204) to detect mouse samples from ABcolonal (Wuhan, China); cleaved PARP1(#9541), cleaved-caspase3 (#9661) and beta-Actin (#4970) from Cell Signaling Technology (Shanghai, China); γH2AX (phospho-Histone H2AX (s139)) (ab81299) from Abcam (Shanghai, China); ki67 (ER1802-31) from Huabio (Hangzhou, China). WIP1 plasmid was kindly provided by Prof. Zhenyu Ju at Hangzhou Normal University. GSK2830371, Diethylnitrosamine (DEN) and TCPOBOP were purchased from Sigma-Aldrich (Shanghai, China). Olaparib (HY-10162) and Veliparib (HY-10129) were purchased from MedChemExpress (Shanghai, China). Other reagents and chemicals don’t list here are commercially available.

### siRNAs and plasmids transfection

siRNAs mentioned in this article were synthesized by Gene Pharma Company (Shanghai, China), and transfected into cells with Lipofectamine™ RNAiMAX transfection reagent (Thermo Fisher Scientific) at a final concentration of 20-50 nM. All sequences of siRNAs used were listed in Additional file 1: Table S1.

For plasmid transfection, cells were seeded overnight in 6 well plates, 2 μg of plasmids were transfected with X-tremeGENE HP DNA Transfection Reagent (Roche Applied Science, Shanghai, China). The mock vector was used as the negative control. Cells were harvested for indicated analysis after 48–72 h later.

### Lentivirus infection

To consistently knockdown WIP1, cells were seeded overnight in 6-well plates and infected with lentivirus containing pLKO.1-scramble (shNC) or pLKO.1-shWIP1 (shWIP1). Stable cells were screened by puromycin. Knockdown of WIP1 was verified by Western blotting, and the constructed stable cells were sent out for cell proliferation in vitro and in vivo*,* respectively. The sequences of primers used were listed in Additional file 1: Table S2.

### Cell growth assay

Cell growth assay was applied with the CellTiter 96® AQueous Non-Radioactive Cell Proliferation Assay kit (Promega, Beijing, China). Briefly, 3,000 PLC/PRF/5 cells or 1000 Hepa1-6 cells per well were seeded into a 96-well plate overnight and treated as indicated, and the MTS reagents from the kit were added to each well. Cell viability was measured following the manufacturer’s instruction. Samples were prepared in triplicates, and the cell viability was determined as the mean ± s.d.

### Apoptosis detection

Cell apoptosis was measured by flow cytometry and western blotting. The FITC‐annexin V and propidium iodide (PI) double staining apoptosis kit (70-AP101, LIANKE BIOTECH, Hangzhou, China) was applied for flow cytometer analysis. Cells treated as indicated for 24–72 h were harvested by trypsin and re-suspended in 500 μl 1 × binding buffer. 5 μl of Annexin V-FITC and 10 μl of PI staining reagent were then added to the cell suspension and incubated for 15 min at room temperature (RT). The samples were analyzed by BD FACSCalibur™ flow cytometer (BD Biosciences). The percentage of apoptotic cells in each group was shown as mean ± SD in the histograms.

### Colony formation assay

Stable knockdown cells were screened by puromycin before colony-formation assays with monolayer cultures. PLC/PRF/5 cells were seeded at 300 cells/well in 6 well plates. Hepa1-6 cells were seeded at 150 cells/well in 6 well plates. After 14 days of culture, cell colonies were counted after staining with 0.5% crystal violet.

### Western blotting

HCC cells treated as indicated were harvested with Radio immunoprecipitation assay (RIPA) buffer and then were quantitated by BCA protein assay kit (Bio-Rad Laboratories**,** Hercules, CA, USA). Lysates were resolved by SDS-PAGE, transferred to PVDF membrane and incubated with the primary antibodies at 4 °C overnight. The membranes then were washed with TBS-T (1 × TBS with 0.1% of Tween-20) and incubated with HRP-conjugated second antibodies (111‐035‐003, Jackson Immuno Research, USA) at RT for 2 h. Finally, the membranes were tested with FDbio- Femto ECL (FD8030, Fudebio, Hangzhou, China), and pictures were processed with Amersham Imager 600 system (GE Healthcare Life Sciences, Shanghai, China).

### Immunohistochemistry (IHC) staining

Formalin-fixed and paraffin-embedded mice liver tissue sections were first sent for hematoxylin–eosin (HE) staining and subsequently immunostained with anti-ki67 antibody using microwave antigen retrieval in 0.01 M pH6.0 citrate buffer. After washing, the signal was detected using a suitable HRP-labeled second antibody with DAB as the chromagen (Dako, Denmark).

### Comet assay

After WIP1 inhibition, single cell suspension was prepared and mix with low melting point agarose at 37 °C. And spread the agarose and cell mixture on the pretreated glass slides. Treat with lysate for 2 h at 4 °C. And perform gel electrophoresis and then neutralize the slides. And after the agarose is completely dry, stain with DAPI dyes (Vectorlabs, H-1200, USA) and observe the degree of DNA damage under fluorescent microscope. The CASP Comet Analysis software. TailDNA% was used to calculate the Tail/Head DNA percent of every single cells. and the average Tail/Head DNA percent was shown as mean ± SD. More than 10 cells were analyzed.

### Immunofluorescence and microscopy

Cells were seeded on coverslips overnight and treated as indicated. Briefly, the cells were fixed with cold methanol for 10 min, permeabilized in 0.25% Triton X-100 for 10 min and blotted with 3% BSA (bovine serum albumin; diluted in PBS) for 30 min. The cells were incubated with appropriate primary antibodies diluted with 3% BSA at 4 °C overnight. Then, the coverslips were washed three times with 0.1% PBS-T (PBS with 0.1% Tween-20), incubated with the appropriate secondary antibodies (Goat anti-Rabbit IgG (H + L) High Cross-Adsorbed Secondary Antibody, Alexa Fluor 488, Invitrogen, A-11034, Shanghai, China) for 1 h at RT, washed and sealed with mounting medium including DAPI. Images were captured on microscope (Olympus, Japan).

### Animal studies

#### DEN induced mice liver cancer model

Wip1 KO mice were kindly provided by Prof. Lawrence A. Donehower [28]. The mice were maintained and treated under specific pathogen-free conditions. To induce hepatocellular carcinogenesis, 100 mg/kg DEN was Intraperitoneally (i.p.) injected into 4-weeks-old male mice, and after 2 weeks, 3 mg/kg TCPOBOP (Sigma) was i.p. injected into the mice every other week for 8 times. Ten months after the DEN injection, mice were euthanized. The liver tissues were collected and divided, one half was immediately frozen in liquid nitrogen and stored at − 80 °C until sent for quantitative real-time RT-PCR (qRT-PCR) and western blot analysis, another half was fixed with 4% formaldehyde immediately and sent for HE staining. The numbers of liver tumors (diameter > 2 mm) of each mouse were counted and student’s t-test was performed for statistical analysis.

#### Mice xenograft model

Male C57BL/6 J mice (6–8 weeks of age) were obtained from Shanghai Laboratory Animal Center and housed in the laboratory-animal research center of Zhejiang University. Hepa1-6-shNC or Hepa1-6-ShWIP1 cells were resuspended with PBS, and 1 × 10^6^ of cells were subcutaneous injected into each mouse (5 mice per group). After 7 days, the growth of implanted tumors was monitored using Vernier calipers every 2 days. Tumor volume (cm^3^) = 0.5 × Tumor length × Tumor width^2^. All mice were sacrificed after 17 days.

Male BALB/c nude mice (n = 56, 6–8 weeks of age) were obtained from Shanghai Laboratory Animal Center and housed in the laboratory-animal research center of Zhejiang University. PLC/PRF/5 cells were resuspended with PBS, and 3 × 10^6^ cells were subcutaneous injected into each mouse. After 7 days, mice were randomized divided into 4 groups (n = 7) and oral treated with Blank, GSK2830371 (100 mg/kg), Olaparib (50 mg/kg), or GSK2830371(50 mg/kg) + Olaparib(25 mg/kg), three times a week. For another experiment, 3 × 10^6^ PLC/PRF/5 cells were subcutaneous injected into each mouse. After 7 days, mice were randomized divided into 4 groups (n = 7) and oral treated with Blank, GSK2830371 (100 mg/kg), Veliparib (100 mg/kg), or GSK2830371(50 mg/kg) + Veliparib(50 mg/kg), three times a week. For each experiment, the growth of implanted tumors was monitored using Vernier calipers three times a week: Tumor volume (cm^3^) = 0.5 × Tumor length × Tumor width^2^. All mice were sacrificed after 23 days.

### Bioinformatics analysis

To identify the association of WIP1 expression with DNA damage response, global gene expression profiles in paired human HCC tissues was obtained from the GEO database (GSE57957), and was analyzed with Gene Set Enrichment Analysis (GSEA) using GSEA 3.0 software (http://www.broadinstitute.org/gsea/), the Gene Set of DNA double-strand break response and Mismatch Repair from MsigDB was employed for GSEA [29]. And the survival analysis and correlation analysis were performed via GEPIA2.0 (http://gepia2.cancer-pku.cn/). The TMB data (Tumor mutation burden) of HCC patients was downloaded from TCGA database, and was calculated via maftools R packge [30].

### Statistical analysis

An independent Student’s t-test was performed to analyze the assay results. Pearson analysis was performed to analyze the correlation. *p* value < 0.05 was considered statistically significant. Results are expressed as mean ± SD as indicated. All experiments were repeated at least three times.

## Results

### High expression of WIP1 correlates with a poor prognosis in HCC

**Fig. 1:**
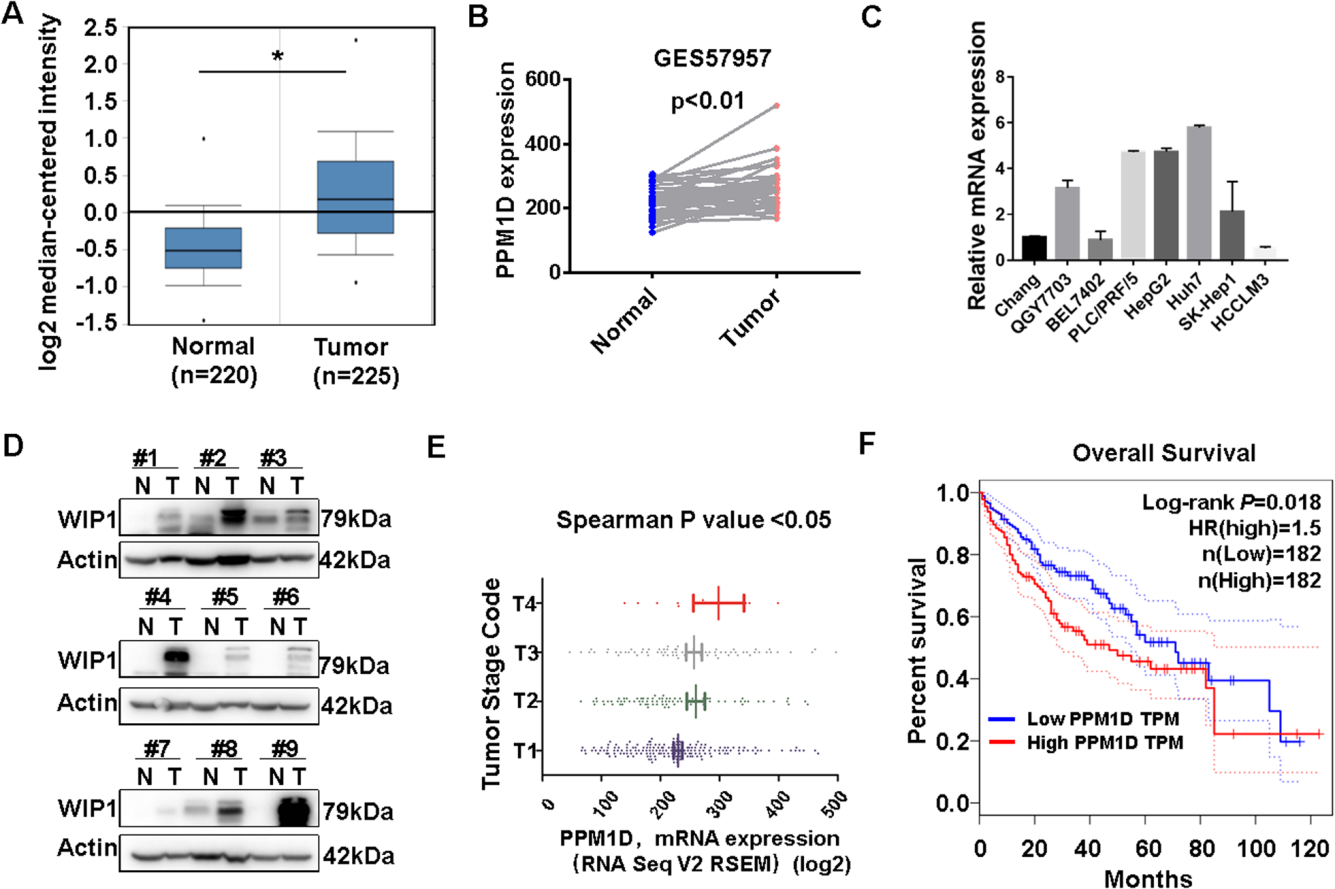
High expression of WIP1 correlates with a poor prognosis in HCC. **A** WIP1 mRNA expression of normal liver tissues and hepatocellular carcinoma of Roessler Liver 2 cohort in Oncomine database (t-test; *p* < 0.01). **B** Expression of WIP1 mRNA in 37 pairs of liver cancer tissues and adjacent non-tumor tissues from GEO data sets GES57957 (Paired t test; *p* < 0.01). **C** The mRNA expression levels of WIP1 in liver cancer cell lines and normal liver cell line (Chang) was detected via qRT-PCR. **D** Expression of WIP1 protein in 9 pairs of liver cancer tissues and adjacent non-tumor tissues was analyzed by Western blotting. **E** The expression of WIP1 mRNA levels in different American Joint Committee on Cancer Tumor Stage Code of TCGA. (Spearman *p* value < 0.05). **F** The impact of WIP1 mRNA expression on overall survival (OS) was analyzed by Kaplan–Meier survival curve (patients were grouped based on median WIP1 mRNA expression)

To determine whether WIP1 is associated with HCC development, we firstly compared the WIP1 mRNA expression level in normal liver and hepatocellular carcinoma tissues. Analysis of multiple microarray data sets in the Oncomine (www.oncomine.org) confirmed that WIP1 mRNA was significantly increased in HCC tissues compared to normal liver tissues (Fig. 1A, *p* < 0.01). In addition, WIP1 mRNA expression in HCC tissues (data from GSE57957) was significantly increased compared to corresponding paired noncancerous tissues (Fig. 1B, *p* < 0.01). Furthermore, the WIP1 mRNA expression in most of the liver cancer cell lines was higher than normal liver cell line (Fig. 1C). Consistently, up-regulated WIP1 protein expression was confirmed in most human HCC tissues (Fig. 1D). Moreover, a higher level of WIP1 mRNA was detected in patients with higher tumor degree (www.cbioprotal.org) (TCGA database, Fig. 1E). And up-regulated WIP1 was associated with shortened patient overall survival (OS) (Fig. 1F, HR = 1.5, Log rank *p* = 0.018). Collectively, these data suggested that WIP1 is up-regulated in HCC, and high expression of WIP1 correlates with a poor prognosis.

### Suppression of WIP1 inhibits proliferation of HCC cells in vitro

**Fig. 2:**
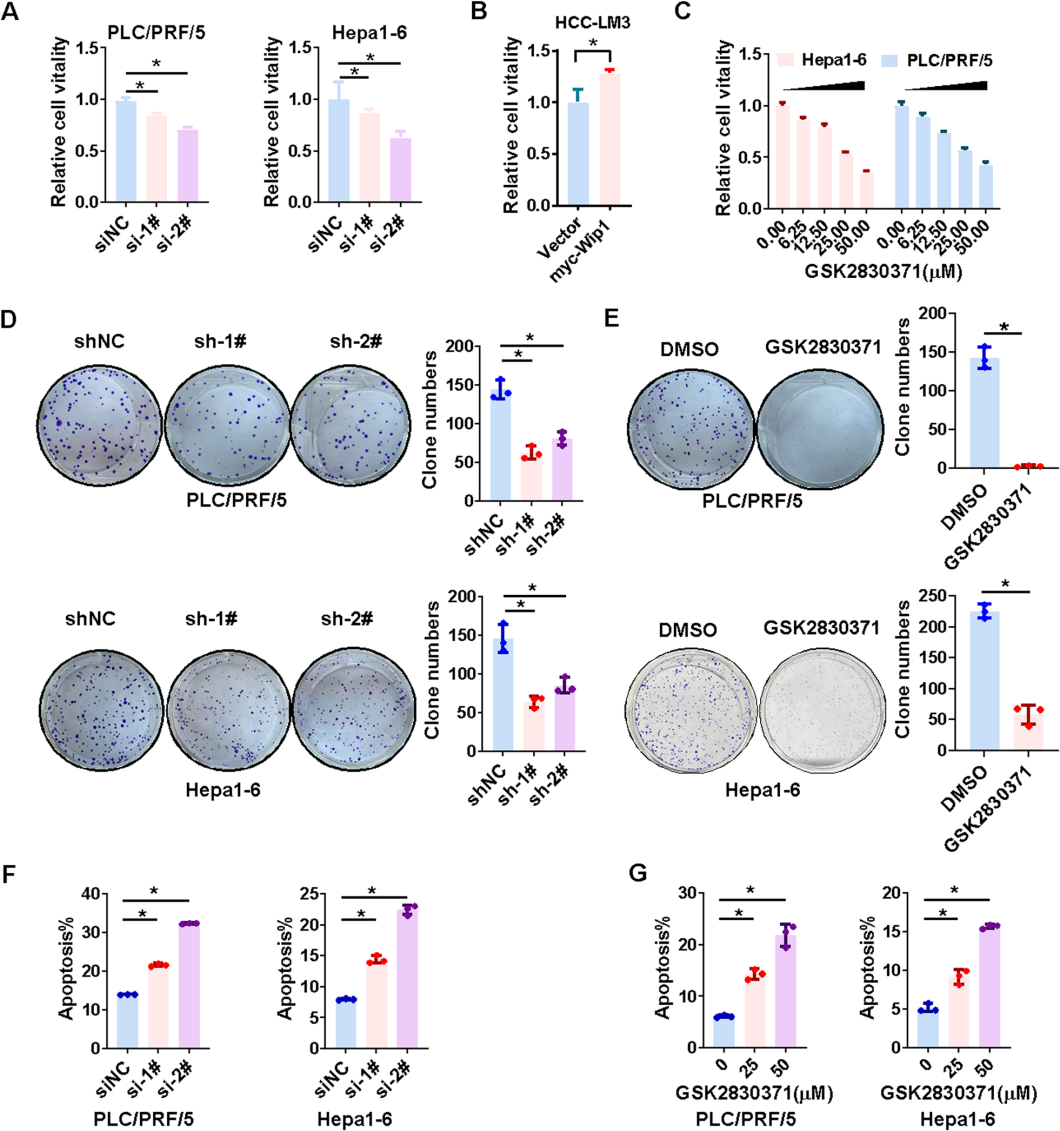
Suppression of WIP1 inhibits proliferation of HCC cells in vitro. **A** Cell viability of PLC/PRF/5 and Hepa1-6 cells with or without WIP1 knockdown by siRNAs was measured with MTS assay. **B** Cell viability of HCC-LM3 cells with ectopic overexpression of WIP1 was measured with MTS assay. **C** Cell viability of PLC/PRF/5 and Hepa1-6 cells treated with WIP1 inhibitor GSK2830371 with indicated concentrations for 72 h was measured with MTS assay. **D** Representative image of colony formation and quantitative analysis of colony numbers of PLC/PRF/5 and Hepa1-6 cells with WIP1 stable knockdown with shRNA. **E** Representative image of colony formation and quantitative analysis of colony numbers of PLC/PRF/5 and Hepa1-6 cells with GSK2830371(12.5 μM, 24 h). **F** The apoptosis of HCC cells with or without WIP1 knockdown with siRNA was assessed via flow cytometry with PI and annexin V–FITC double staining. **G** The apoptosis of HCC cells with or without GSK2830371 treatment for 72 h was assessed via flow cytometry with PI and annexin V–FITC double staining

Next, we want to investigate the role of WIP1 in hepatocellular carcinogenesis. Firstly, knockdown of WIP1 could significantly inhibit HCC cell growth (Fig. 2A, and Additional file 1: Fig. 1A). On the other hand, ectopic expression of WIP1 predominantly increased HCC cell growth (Fig. 2B, and Additional file 1: Fig. 1B). As an allosteric inhibitor of WIP1, GSK2830371 interacts with a ‘flap’ subdomain near the Wip1 catalytic site and thereby confers selectivity over other phosphatases [31]. Indeed, GSK2830371 inhibited HCC cell proliferation as well (Fig. 2C). Similarly, WIP1 knockdown or inhibition with GSK2830371 attenuated clone formation of HCC cells (Fig. 2D, E, Additional file 1: Fig. 1C). In addition, suppression of WIP1 expression or activity could induce apoptosis in HCC cells (Fig. 2F, G, and Additional file 1: Fig. 1D-F). In summary, suppression of WIP1 reduces cell proliferation and induces apoptosis in HCC cells.

### WIP1 suppression inhibits HCC development in vivo

**Fig. 3:**
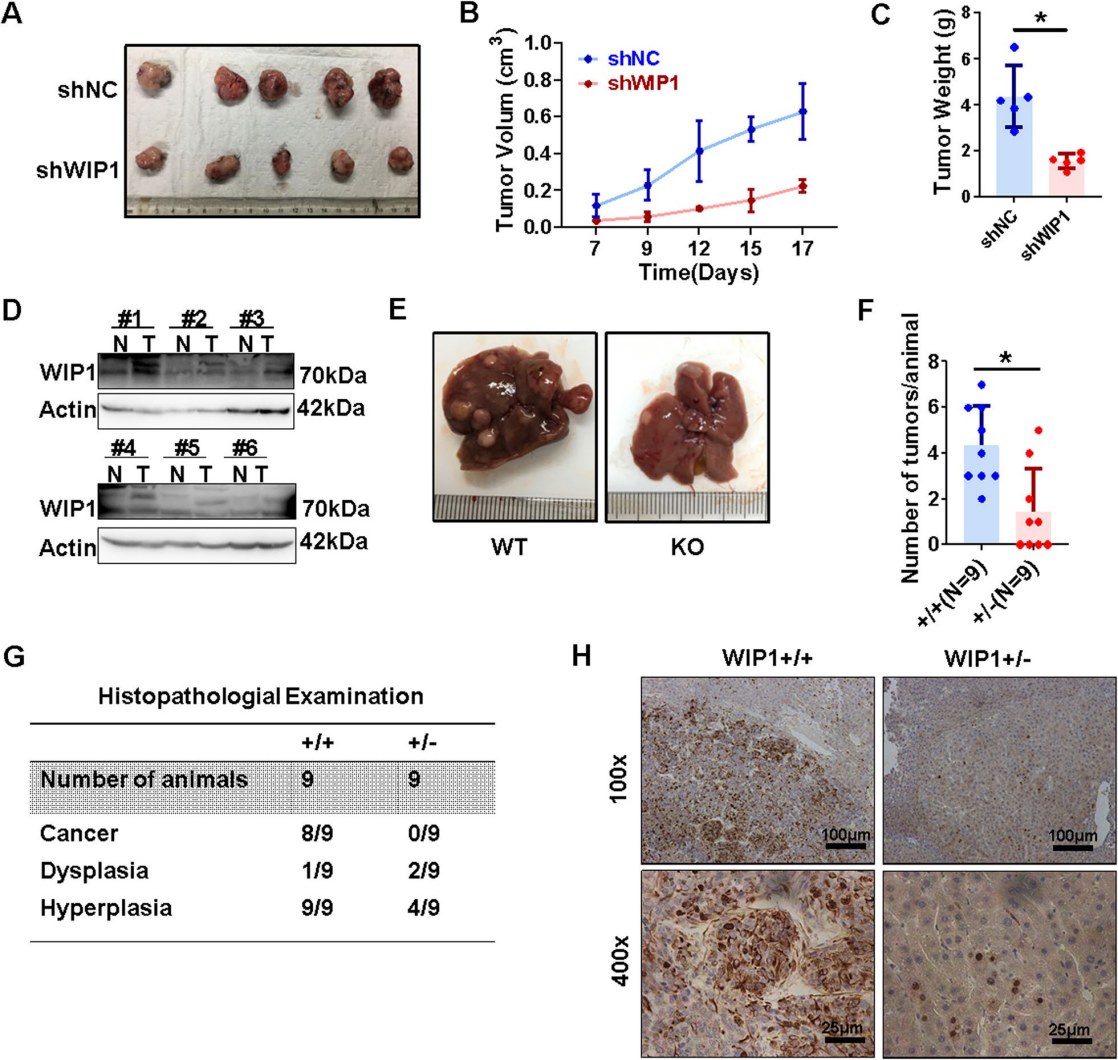
WIP1 suppression inhibits HCC development in vivo. Xenograft model (n = 5 per group) was performed with WIP1 stably knockdown Hepa1-6 cells (Hepa1-6-shWIP1), controlled with Hepa1-6-shNC stable cells. And tumor pictures (**A**), tumor growth curve (**B**) and tumor weight (**C**) were shown respectively. **D** WIP1 protein level in 6 pairs of DEN-induced mice liver cancer tissues and adjacent non-tumor tissues was analyzed by Western blotting. **E** Representative macroscopic images of WIP1 wild-type (+ / +) or knockout (+ / −) mice with DEN-induced HCC. **F** The average number of tumors per mouse in DEN-induced HCC (size > 2 mm) in WIP1 wild-type (+ / +) or knockout (+ / −) mice. **G** The histopathological examination of wild-type (+ / +) or knockout (+ / −) mice liver tissues. **H** The ki-67 immunohistochemical staining of wild-type (+ / +) or knockout (+ / −) mice liver tissue

To further explore the relevance of WIP1 to HCC development in vivo*,* the xenograft mice model was applied. Compared to stable Hepa1-6-shNC cells, the growth of Hepa1-6-shWIP1 cells-formed tumors were significantly impaired (Fig. 3A–C). In addition, a widely used DEN-induced hepatocellular carcinogenesis mice model was adopted to evaluate the role of WIP in HCC [32, 33]. Firstly, we confirmed that WIP1 protein expression was up-regulated in mouse liver cancer tissues compared with paired normal tissues (Fig. 3D). Furthermore, consistent with in vitro results, DEN-induced WIP1 knockout ( ±) mice showed a significant decreased number of liver tumors compared to wild type mice (Fig. 3E–G, Additional file 1: Fig. 2A-B). And compared with tumors from control mice, WIP1 deficient decreased expression of Ki67 (Fig. 3H), which indicated impaired cell proliferation in vivo. These data confirmed that suppression of WIP1 inhibits HCC development in vivo.

### WIP1 inhibition disrupts DNA damage repair by increasing H2AX phosphorylation

**Fig. 4:**
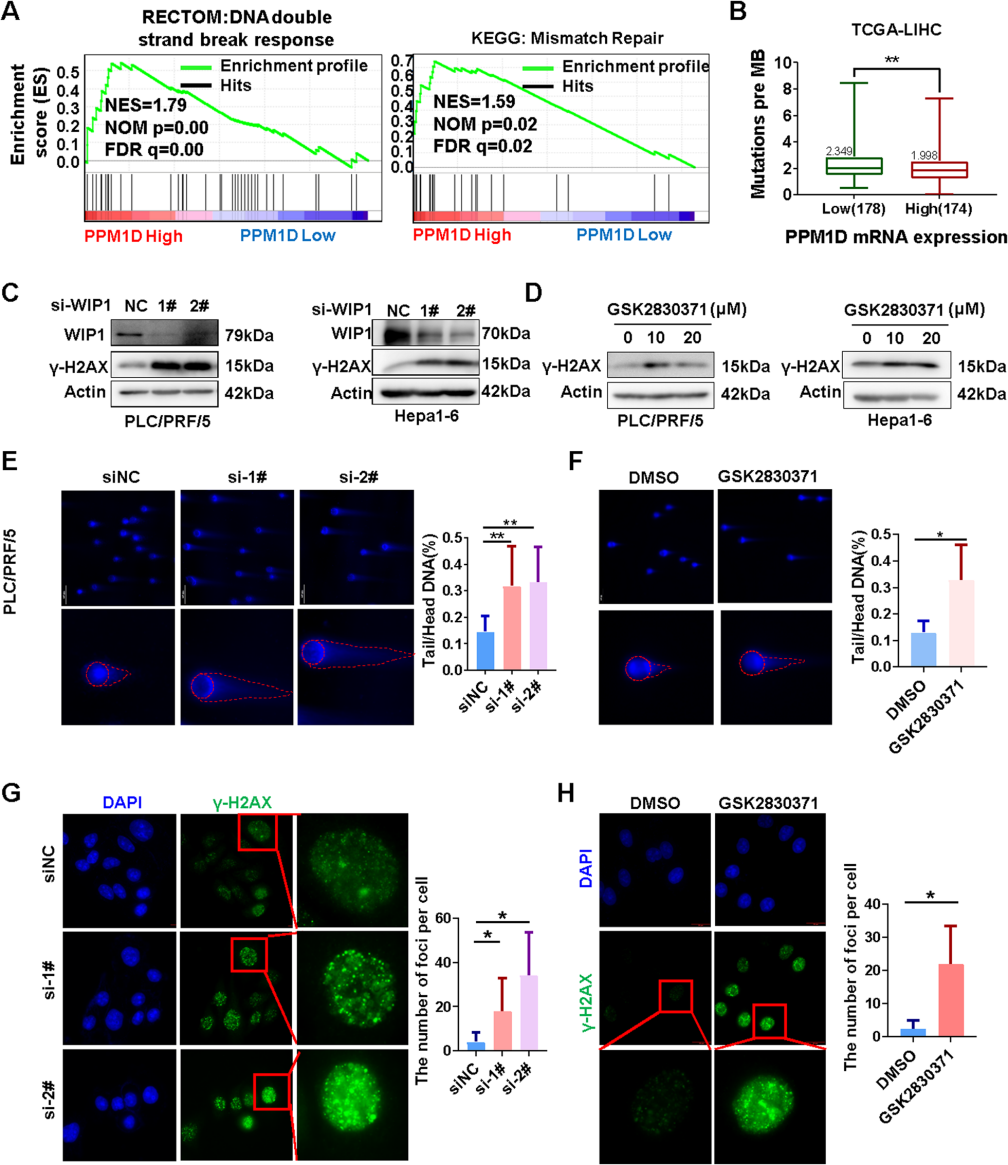
WIP1 inhibition disrupts DNA damage repair by increasing H2AX phosphorylation. **A** Gene set enrichment analysis (GSEA) of the gene expression profiles in WIP1 high expression and WIP1 low expression human liver cancer tissues. Red indicates WIP1 high expression; blue indicated WIP1 low expression. **B** Tumor mutation burden (TMB) was compared between WIP1 high and low expression liver cancer tissues from TCGA database. The phosphorylation of H2AX at S139 (γH2AX) was measured via Western blotting in HCC cells after WIP1 knockdown (**C**) or GSK2830371 inhibition (**D**). The Comet assay was performed to detect the DNA double strand break of PLC/PRF/5 cells after WIP1 knockdown (**E**) or GSK2830371 inhibition (25 μM, 48 h) (**F**). CASP software was used to calculate Tail/Head DNA percent of every single cell. The foci of γH2AX was measured via immunofluorescence to evaluated the DNA damage levels of PLC/PRF/5 cells after WIP1 knockdown (**G**) or GSK2830371 inhibition (25 μM, 48 h) (**H**). And the numbers of foci per cell were counted and shown

To investigate how WIP1 promotes HCC development, we firstly analyzed the gene expression files from GSE57957 by gene sets enrichment analysis (GSEA). We found that up-regulated WIP1 was associated with activated DNA double-strand break response and mismatch repair signature (Fig. 4A). Consistently, the high WIP1 mRNA level was positively correlated with the expression of DNA double-strand break response and mismatch repair signature in TCGA LIHC database (Additional file 1: Fig. 3A and 3B). Meanwhile, we confirmed that DNA double-strand break response and mismatch repair signature were up-regulated in HCC tumor tissues compared to paired non-tumor tissues (Additional file 1: Fig. 2C and 2D). The up-regulated signature was also associated with shortened overall survival (OS) of HCC patients (Additional file 1: Fig. 3E and 3F). Moreover, the up-regulated WIP1 mRNA expression was correlated with a lower tumor mutation burden (TMB) in TCGA LIHC database (Fig. 4B). These results indicated that up-regulated WIP1 expression could enhance DNA damage repair to promote HCC development.

Previous studies have found that WIP1 plays critical roles in the regulation of DNA damage repair through directly dephosphorylating several DNA damage repair associated proteins, including p53, H2AX [13], p38 MAPK [14, 15], and chk1. Here, we found that the phosphorylation of H2AX (gamma-H2AX, γH2AX), but not the phosphorylation of mTOR, p53 and p38 MAPK (Additional file 1: Fig. 3G-H), was regulated by WIP1 in HCC cells. The level of γH2AX was up-regulated in HCC cells with WIP1 knockdown or inhibition (Fig. 4C, D). And increasing DNA damage in HCC cells with WIP1 knockdown or inhibition was found via comet assay (Fig. 4E, F, and Additional file 1: Fig. 4A), which was further confirmed via immunofluorescence staining of γH2AX foci in HCC cells with WIP1 knockdown or inhibition (Fig. 4G, H, and Additional file 1: Fig. 4B-C). In summary, suppression of WIP1 could abrogate DNA damage repair in HCC cells.

### WIP1 and PARP inhibition pronounced DNA damage

**Fig. 5:**
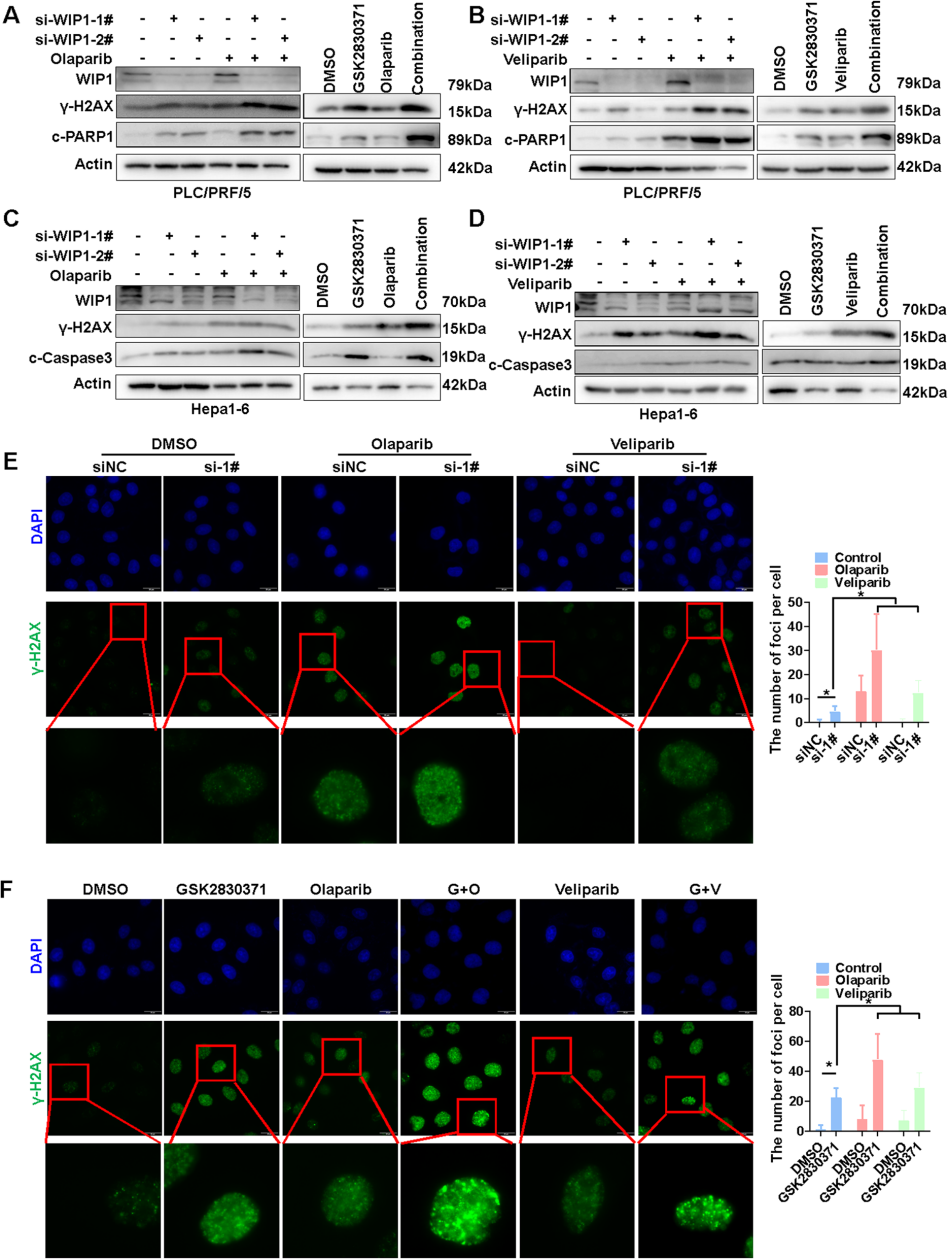
WIP1 and PARP inhibition pronounced DNA damage. **A** γH2AX and cleaved-PARP1 (C-PARP1) were measured via Western blotting in PLC/PRF/5 cells after WIP1 knockdown or inhibition combined with Olaparib (50 μM, 24 h) treatment. **B** γH2AX and C-PARP1 was measured via Western blotting to evaluated the DNA damage levels and apoptosis of PLC/PRF/5 cells after WIP1 knockdown or inhibition combined with Veliparib (50 μM, 24 h) treatment. **C** γH2AX and C-Caspase3 was measured via Western blotting in Hepa1-6 cells after WIP1 inhibition combined with Olaparib (50 μM, 24 h) treatment. **D** γH2AX and c-PARP1 was measured via Western blotting to evaluated the DNA damage levels and apoptosis of Hepa1-6 cells after WIP1 inhibition combined with Veliparib (50 μM, 24 h) treatment. **E** The foci of γH2AX was measured via immunofluorescence in PLC/PRF/5 cells after WIP1 knockdown combined with Olaparib (50 μM) or Veliparib (50 μM) treatment for 24 h. And the numbers of foci per cell were counted and shown. **F** The foci of γH2AX was measured via immunofluorescence to evaluated the DNA damage levels of PLC/PRF/5 cells after GSK2830371(25 μM) combined with Olaparib (50 μM) or Veliparib (50 μM) treatment for 24 h. And the numbers of foci per cell were counted and shown

Inhibition of poly-(ADP-ribose) polymerase (PARP), a key enzyme in base excision repair, efficiently kills cancer cells with defective Homologous recombination (HR) in BRCA1/2 deficient cancer, which turned as synthetic lethal due to enhanced DNA damage [34]. Upon these findings, PARP inhibitors including olaparib are now clinically used to treat BRCA1/2-deficient breast and ovary cancers [34]. Since γH2AX plays an important role in HR, we want to know whether suppression of WIP1 together with PARP inhibition could be synthetic lethal in HCC cells. Indeed, combined treatment with PARP inhibitors (olaparib and veliparib) and WIP1 knockdown or inhibition increased the level of γH2AX in HCC cells (Fig. 5A–D). And the increasing DNA damage was also confirmed via immunofluorescence staining of γH2AX foci in HCC cells with WIP1 knockdown or inhibition combined with PARP inhibitors (Fig. 5E, F, and Additional file 1: Fig. 5A and 5B). The above findings suggested that suppression of WIP1 synergy with PARP inhibition to enhance DNA damage in HCC cells.

### WIP1 and PARP inhibition induce synthetic lethality in HCC both in vitro and in vivo

**Fig. 6:**
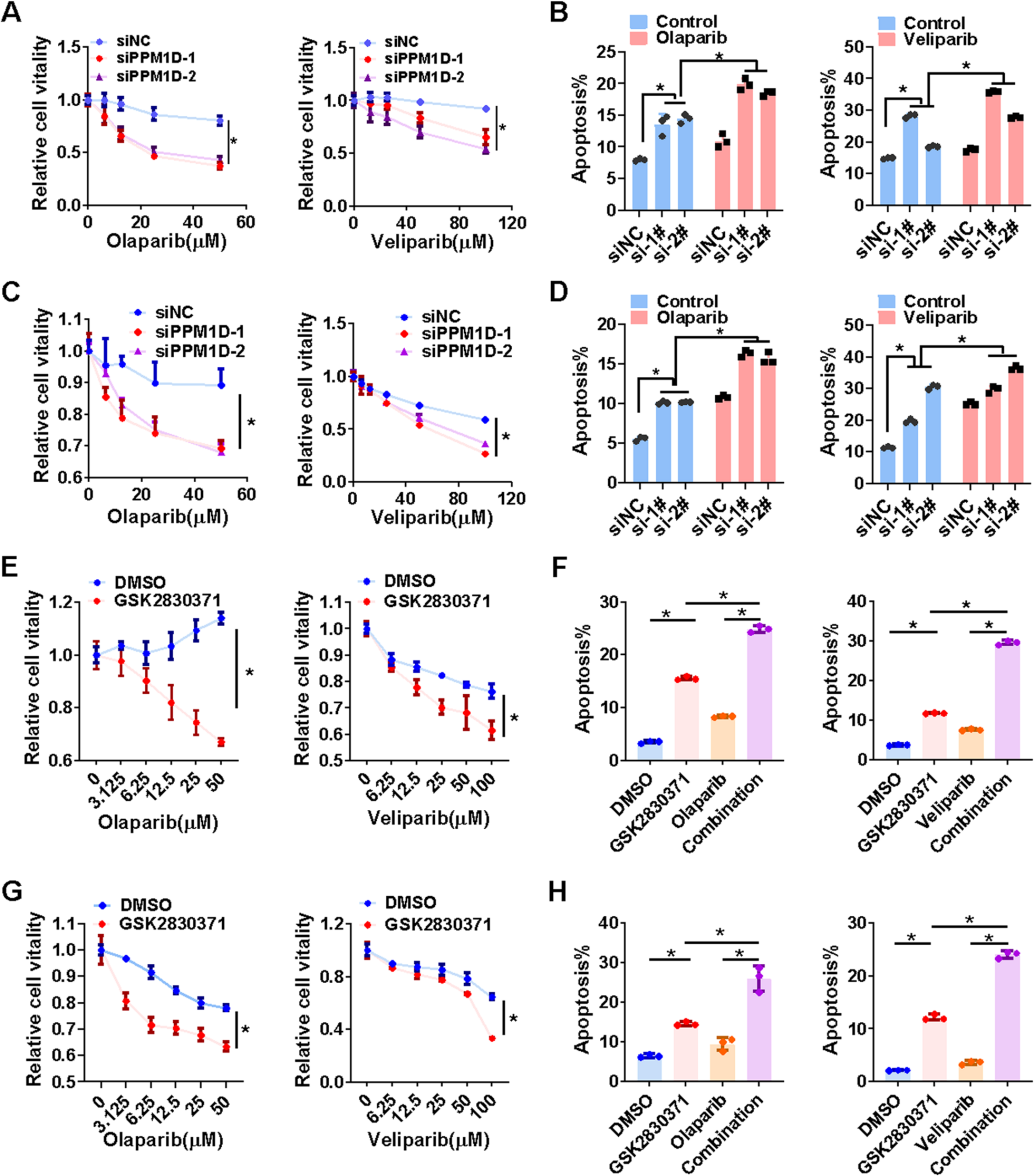
WIP1 and PARP inhibition confers HCC cells synthetic lethal in vitro. **A** Cell viability of Hepa1-6 cells after Olaparib (48 h) or Veliparib (48 h) treatment with or without WIP1 knockdown was measured with MTS assay. **B** The apoptosis of Hepa1-6 cells after Olaparib (50 μM, 48 h) or Veliparib treatments with or without WIP1 knockdown was measured via flow cytometry with PI and annexin V–FITC double staining. **C** Cell viability of PLC/PRF/5 cells after Olaparib or Veliparib treatment with or without WIP1 inhibitor GSK2830371 was measured with MTS assay. **D** The apoptosis of PLC/PRF/5 cells after Olaparib or Veliparib treatment with or without WIP1 inhibitor GSK2830371 was measured via flow cytometry. **E** Cell viability of Hepa1-6 cells after Olaparib or Veliparib treatments with or without GSK2830371 treatment was measured with MTS assay. **F** The apoptosis of Hepa1-6 cells after Olaparib or Veliparib treatments with or without GSK2830371 treatment was measured via flow cytometry. **G** Cell viability of PLC/PRF/5 cells after Olaparib or Veliparib treatments with or without GSK2830371 treatment was measured with MTS assay. **H** The apoptosis of PLC/PRF/5 cells after Olaparib or Veliparib treatments with or without GSK2830371 treatment was measured via flow cytometry

**Fig. 7:**
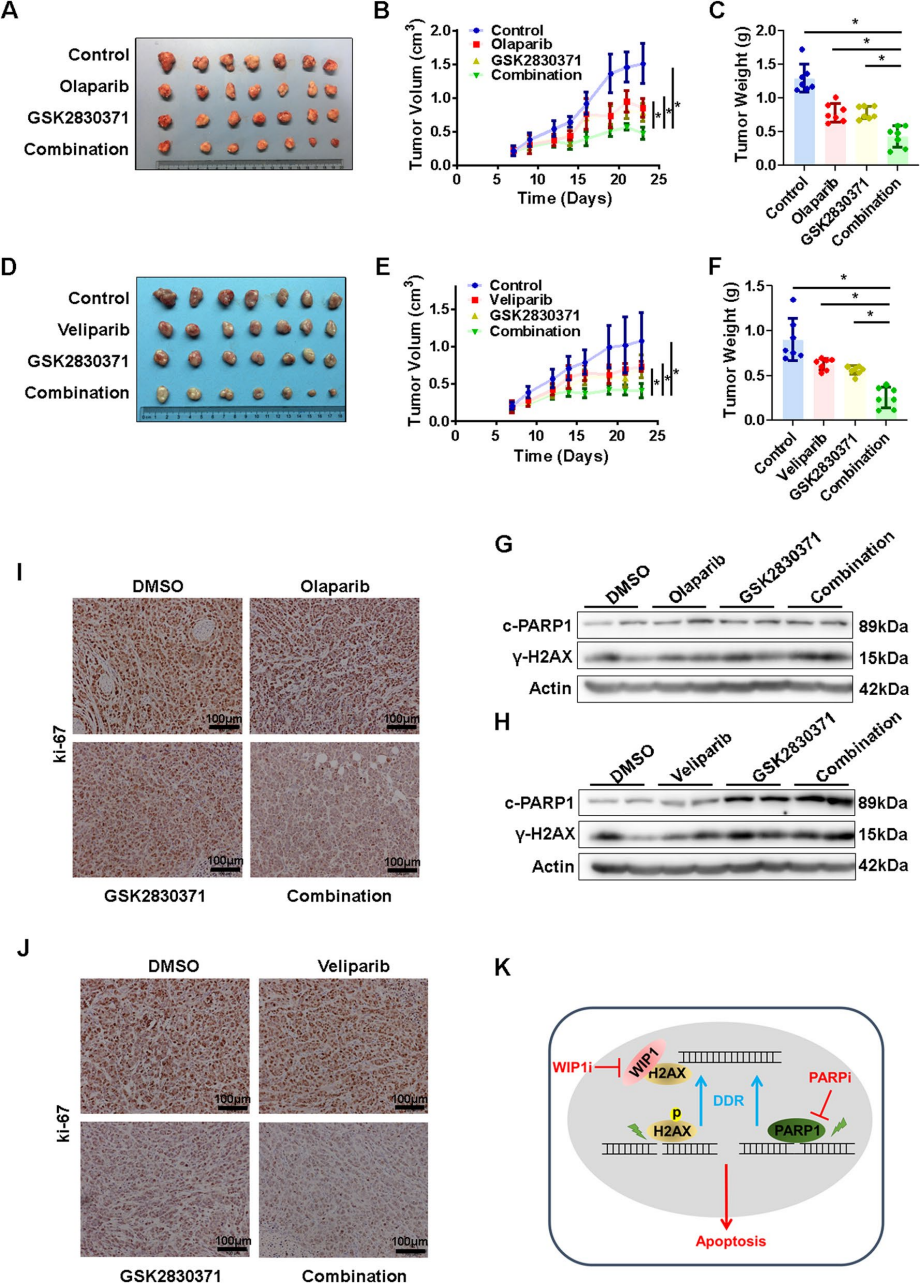
WIP1 and PARP inhibition induce synthetic lethality of HCC cells in vivo. Xenograft model (n = 7 per group) was generated by subcutaneous inoculation of PLC/PRF/5 cells. Mice were then treated with Olaparib and GSK2830371 as indicated. Tumor pictures (**A**), tumor growth curve (**B**) and tumor weight (**C**) were summarized and shown respectively. Veliparib and GSK2830371 combination treatment experiment was performed as above, and tumor pictures (**D**), tumor growth curve (**E**) and tumor weight (**F**) were summarized and shown respectively. C-PARP1 and γH2AX in tumor tissues were evaluated by Western blotting, Olaparib and GSK2830371 combination in (**G**), Veliparib and GSK2830371 combination in (**H**). **I**–**J** The ki-67 immunohistochemical staining of tumor tissues with indicated treatment was shown. **K** Working model. WIP1 functions as a homeostatic regulator during DNA double strand break by de-phosphorylating γH2AX at the end of DNA damage repair. Thus, co-targeting WIP1 and PARP could induce HCC synthetic lethality via disrupting DNA damage repair, which likes the PARPi works in BRCA1/2 deficient cancers

According to the above findings, we explored the synthetic lethal effect of WIP1 and PARP inhibition in HCC. As expected, WIP1 knockdown increased the sensitivity of PARP inhibitors and promoted apoptosis in HCC cells (Fig. 6A–D, Additional file 1: Fig. 6A-D). Consistently, WIP1 inhibition by GSK2830371 also increased the sensitivity of PARP inhibitors and promoted apoptosis in HCC cells (Fig. 6E–H, Additional file 1: Fig. 6E). Furthermore, in line with in vitro findings, the combination of GSK2830371 with PARP inhibitors significantly retarded tumor growth in nude mice xenograft model (Fig. 7A–F). Compared to either single agent administration, more DNA damage, apoptosis, and proliferation attenuation were found after the combined treatment of GSK2830371 and PARP inhibitors (Fig. 7G–J). In summary, the combinational inhibition of WIP1 and PARP could induce synthetic lethality in HCC.

## Discussion

Hepatocellular carcinoma (HCC) is one of the leading causes of cancer deaths worldwide. Understanding the molecular mechanisms of HCC pathogenesis is urgently needed to develop novel clinical strategies. Previous studies have found that WIP1 is up-regulated in HCC, and down-regulated microRNA-29c contributes to its target gene WIP1 high expression ^24^. Consistently, we confirmed high expression of WIP1 in HCC, and further found that WIP1 high expression correlates with a poor prognosis in HCC patients (Fig. 1). Additionally, we found that suppression of WIP1 could remarkably inhibit HCC cell proliferation both in vitro and in vivo via increasing DNA damage (Figs. 2, 3, 4). Moreover, WIP1 deficiency significantly retarded DEN-induced hepato-carcinogenesis (Fig. 3). Thus, WIP1 might work as an oncoprotein in HCC.

As a phosphatase, WIP1 is implicated in DNA damage repair pathways by directly dephosphorylating several proteins including p53 [7], H2AX [13], p38 [14, 15], chk1 [16], and chk2 [35]. Herein, we found that γH2AX, but not the phosphorylation of other DNA damage associated proteins, was increased in HCC cells after WIP1 inhibition (Fig. 4).

Genomic instability is one of the hallmarks of cancer cells, which is associated with a greater propensity to accumulate DNA damage [36]. Hence, DNA damage repair (DDR) signaling is usually pronounced to control the genome integrity in cancer cells. In the process of DNA damage repair, γH2AX plays a crucial role in recruiting DNA damage repair factors such as BRCA1, MRE11/RAD50/NBS1 complex, and 53BP1 to repair damaged DNA [37]. Upon the completion of DNA damage repair, γH2AX needs to be dephosphorylated and removed for checkpoint recovery [38]. Thus the turnover of γH2AX needs to be precisely controlled during the DNA damage repair process. Indeed, WIP1 acts as a checkpoint regulator that could dephosphorylate γH2AX directly and remove γH2AX from chromatin to disassociate the DNA damage repair complex, which promotes repaired cells to re-enter cell cycle arrest [39]. Recent studies revealed that clonal hematopoiesis with the gain of function mutations in WIP1 out competed their wild-type counterparts in *vivo* after exposure to DNA damage stress [40, 41]. Therefore, WIP1 could function as a homeostatic regulator during DNA damage [42]. In line with these findings, our results indicated the expression of WIP1 had positive correlation with DNA damage repair signature in HCC. And suppression of WIP1 induced DNA damage and apoptosis in HCC cells via increasing γH2AX (Fig. 4). Taken together, we hypothesis that WIP1 dephosphorylates γH2AX at the late step of DNA damage repair to remove H2AX from DNA damage site, which would facilitate the repair kinetics in HCC cells.

The PARP family enzymes covalently add Poly(ADP- ribose) (PAR) chains on to target proteins, termed PARylation, which is involved in chromatin modification, DNA damage repair, maintenance of telomeres and so on [43]. PARP1/2 are the best-studied PARP enzyme, PARP1/2 and BRCA inhibition induce cancer cell synthetic lethal via disturbing DNA damage repair [44]. Besides, PARP inhibitors (PARPi) are now used clinically to treat BRCA1/2-deficient breast and ovary cancer [45, 46]. Combinations of PARPi with other drugs are now being intensively investigated to prevent the development of resistance to PARPi and to extend their use beyond BRCA1/2-deficient tumors including HCC [47–49]. The previous report showed that WIP1 deficient cells are more sensitive to PARP inhibition, WIP1 inhibitor and olaparib combination was associated with increased cell death [50]. Consistently, we confirmed that WIP1 suppression together with PARP inhibition induced synthetic lethality in HCC via enhancing DNA damage, and combination of WIP1 and PARP inhibition suppressed HCC cell proliferation significantly both in vitro and in vivo (Figs. 5, 6, 7). These results suggested that in WIP1 high expressed HCC, WIP1 inhibition might extend the PAPRi indication in future.

## Conclusion

WIP1 plays an oncogenic role in HCC development via regulating DNA damage repair. Targeting WIP1 alone or in combination with PARPi may provide a novel strategy for HCC precise management (Fig. 7K).

## Supplementary Information

**Additional file 1.** Additional results, figures, protocols and tables. Supplementary Figures S1-S6 show additional data related to the results shown in the main figures. **Supplemental Figure 1**. WIP1 inhibition suppresses HCC cell proliferation in vitro. **Supplemental Figure 2**. WIP1 inhibition suppresses HCC development in vivo. **Supplemental Figure 3**. WIP1 inhibition disrupts DNA damage repair by increasing H2AX phosphorylation. **Supplemental Figure 4**. WIP1 inhibition disrupts DNA damage repair by increasing H2AX phosphorylation. **Supplemental Figure 5**. WIP1 and PARP inhibition enhances DNA damage. Supplemental Figure 6. WIP1 and PARP inhibition induce synthetic lethality of HCC cells in vitro. **Table S1**. siRNA sequences used for knockdown. **Table S2**. shRNA sequences used for knockdown.

## Data Availability

Available upon request.

## Abbreviations

HCC: Hepatocellular carcinoma
WIP1: Wild-type p53-induced phosphatase 1
DEN: Diethylnitrosamine
RIPA: Radio immunoprecitation assay
IHC: Immunohistochemistry
HE: Hematoxylin–eosin
GSEA: Gene set enrichment analysis
TMB: Tumor mutation burden
OS: Overall survival
CCLE: Cancer cell line encyclopedia
PI: Propidium iodide
C-PARP1: Cleave-PARP1
C-Caspase3: Cleave-caspase3
WT: WIP1 wild-type
LW/BW: Liver weight/body weight ratio
γH2AX: Phosphorylated H2AX at Ser139
PARP: Poly-(ADP-ribose) polymerase
HR: Homologous recombination
DDR: DNA damage repair
PARPi: PARP inhibitors
