# Supplementary material for: Co-targeting WIP1 and PARP induces synthetic lethality in hepatocellular carcinoma
Source: Cell Commun Signal. 2022 Mar 28;20:39. doi: 10.1186/s12964-022-00850-2 (PMC8962187; doi:10.1186/s12964-022-00850-2)
Supplement: Supplementary file 2 — Additional file 1. Additional results, figures, protocols and tables. Supplementary Figures S1-S6 show additional data related to the results shown in the main figures. Supplemental Figure 1. WIP1 inhibition suppresses HCC cell proliferation in vitro. Supplemental Figure 2. WIP1 inhibition suppresses HCC development in vivo. Supplemental Figure 3. WIP1 inhibition disrupts DNA damage repair by increasing H2AX phosphorylation. Supplemental Figure 4. WIP1 inhibition disrupts DNA damage repair by increasing H2AX phosphorylation. Supplemental Figure 5. WIP1 and PARP inhibition enhances DNA damage. Supplemental Figure 6. WIP1 and PARP inhibition induce synthetic lethality of HCC cells in vitro. Table S1. siRNA sequences used for knockdown. Table S2. shRNA sequences used for knockdown. [file 12964_2022_850_MOESM2_ESM.docx]

Supplemental Figures and Tables


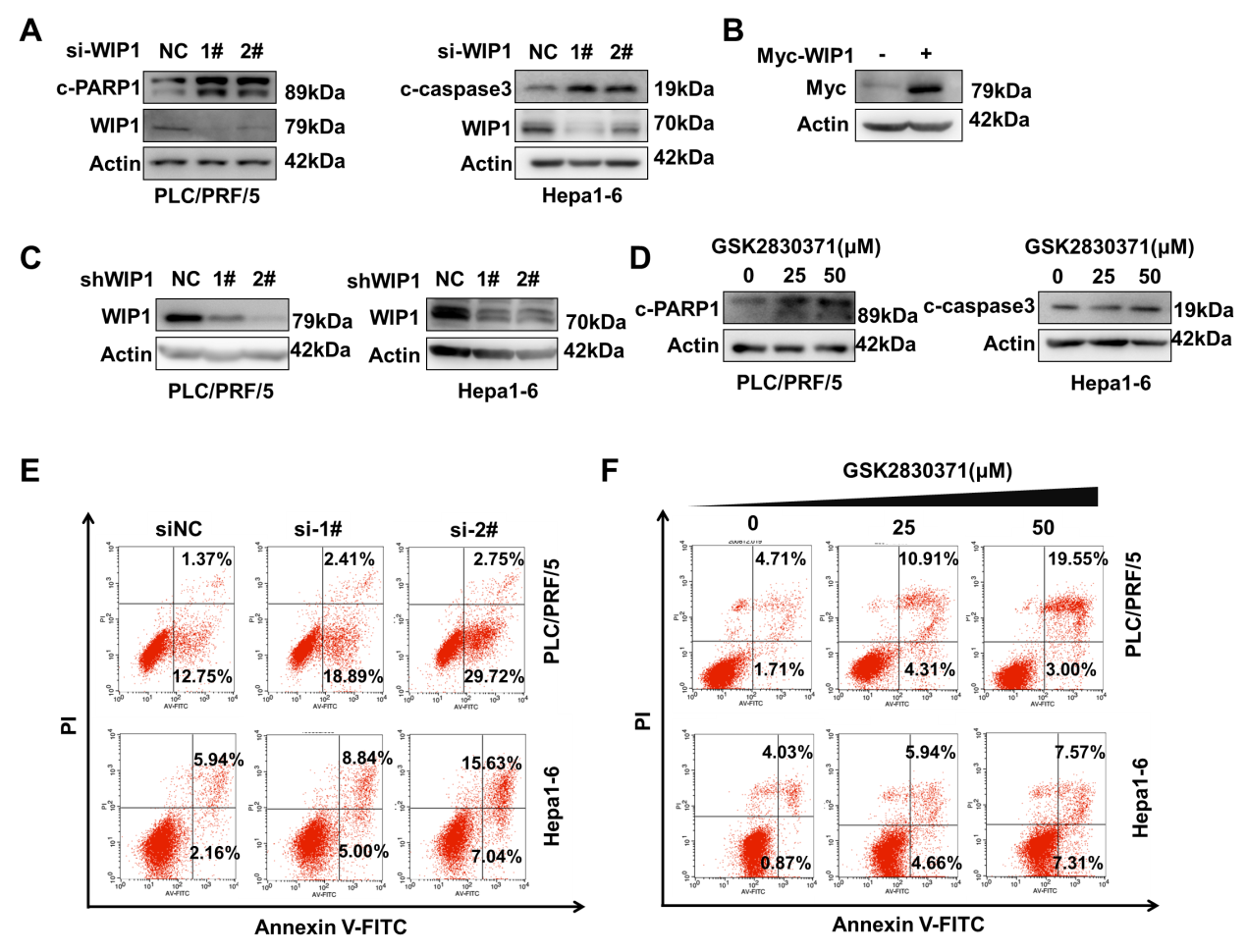


**Supplemental Figure 1. WIP1 inhibition suppresses HCC cell proliferation *in vitro*.**

A. The expression of WIP1, Cleave-PARP1 (C-PARP1), Cleaved-Caspase3 (C-caspase3) in HCC cells with or without WIP1 knockdown were measured with western blotting.

B. The expression of Myc-WIP1 in HCC-LM3 cells with Myc-WIP1 transient transfection was detected by western blotting.

C. The expression of WIP1 in HCC cells with stably WIP1 knockdown (shWIP1) was measured with western blotting.

D. The level of C-PARP1 and C-caspase3 in HCC cells with GSK2830371 treatment was measured with western blotting.

The representative apoptosis pictures of PLC/PRF/5 and Hepa1-6 cells with WIP1 knockdown (E) or WIP1 inhibition (F) analyzed with the annexinⅤ-PI double staining assay were shown.


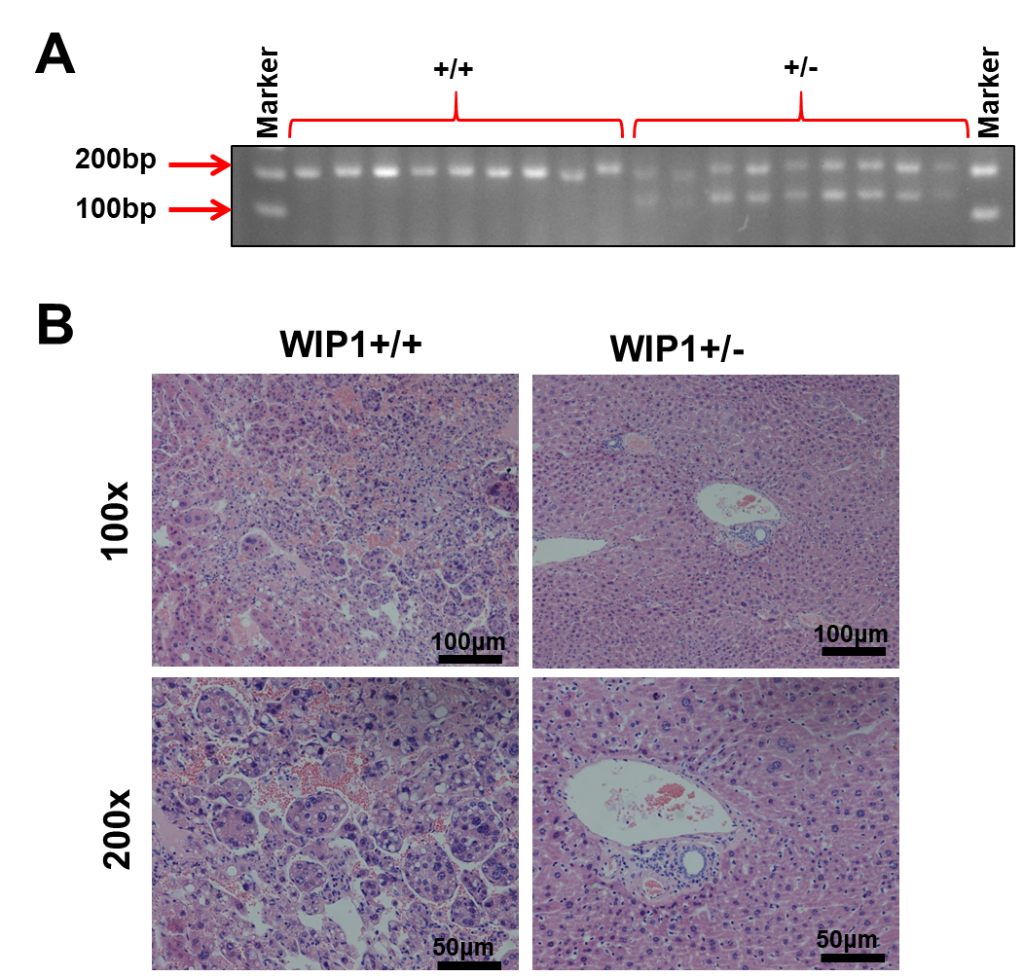


**Supplemental Figure 2. WIP1 inhibition suppresses HCC development *in vivo***

A. Genotypes of WIP1 wildtype (+/+) or knockout (+/-) mice were detected by PCR and DNA gel electrophoresis.

B. Representative H&E staining microscopic images of liver tissues in DEN-induced WIP1 +/+ or +/- mice.


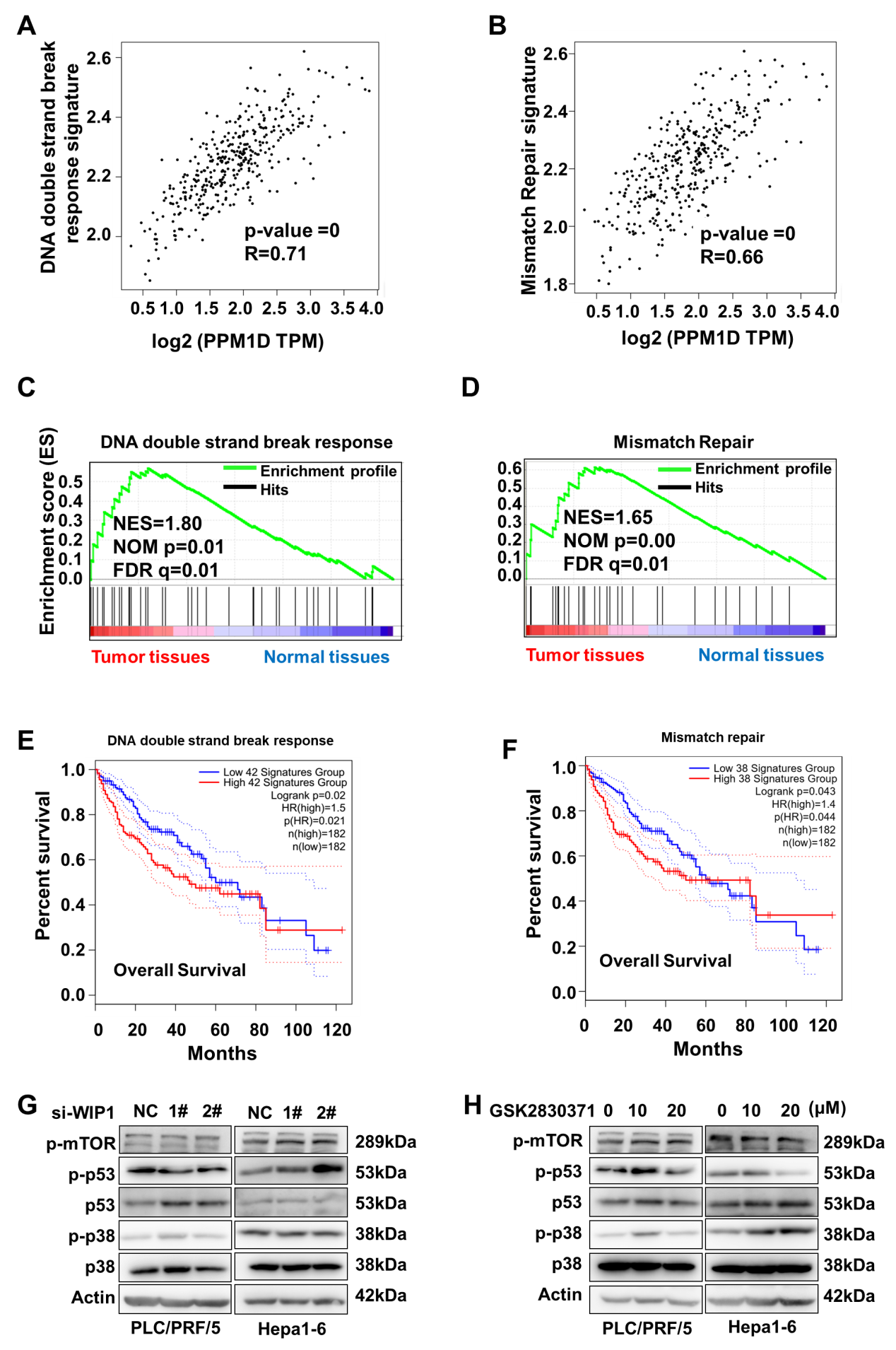


**Supplemental Figure 3. WIP1 inhibition disrupts DNA damage repair by increasing H2AX phosphorylation.**

A. Correlation analysis of WIP1 TPM and double-strand break response signature was analyzed with GEPIA 2.0.

B. Correlation analysis of WIP1 TPM and Mismatch repair signature was analyzed with GEPIA 2.0.

C-D. Gene set enrichment analysis (GSEA) of normal liver tissues compared to liver tumor tissues. Red indicates tumor tissues; Blue indicates normal liver tissues.

E. The impact of double-strand break signature expression on overall survival (OS) of liver cancer patients was analyzed with GEPIA 2.0.

F. The impact of Mismatch repair signature expression on overall survival (OS) of liver cancer patients was analyzed with GEPIA 2.0.

G-H. The p-mTOR, p-p53, and p-p38 levels were measured after WIP1 knockdown (G) or GSK2830371 inhibition (H) in HCC cells via western blotting.


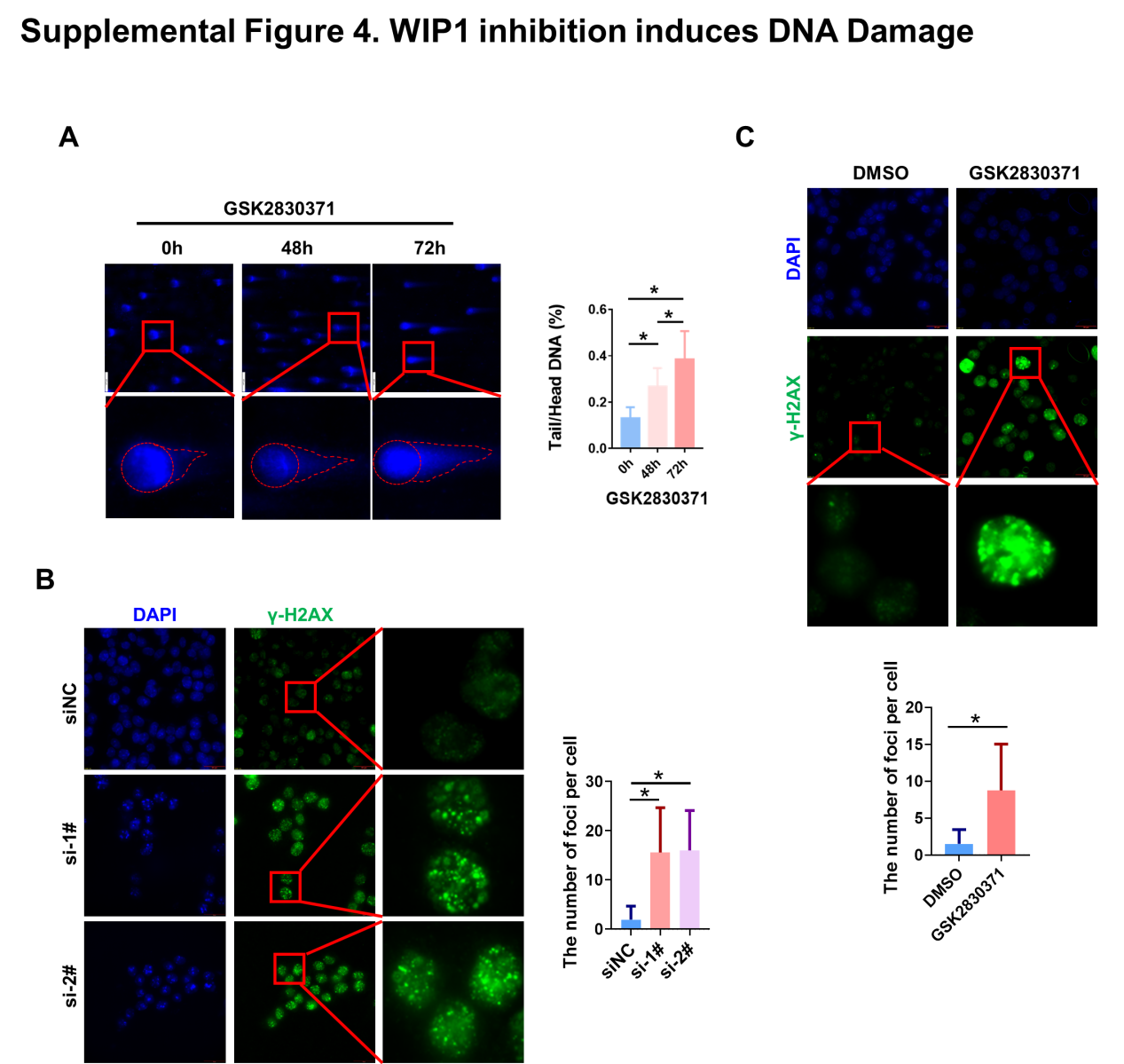


**Supplemental Figure 4. WIP1 inhibition disrupts DNA damage repair by increasing H2AX phosphorylation.**

A. The Comet assay was performed to detect the DNA double-strand break of PLC/PRF/5 cells after GSK2830371 inhibition (25 μM) at different times. CASP software was used to calculate the Tail/Head DNA percent of every single cell, and the average Tail/Head DNA percent was shown as mean ± SD.

B-C. The foci of phosphorylation of H2AX at S139 (γH2AX) was measured via immunofluorescence in Hepa1-6 cells after WIP1 knockdown (B) or GSK2830371 inhibition (C). The numbers of foci were counted, and the average foci number per cell was shown as mean ± SD.


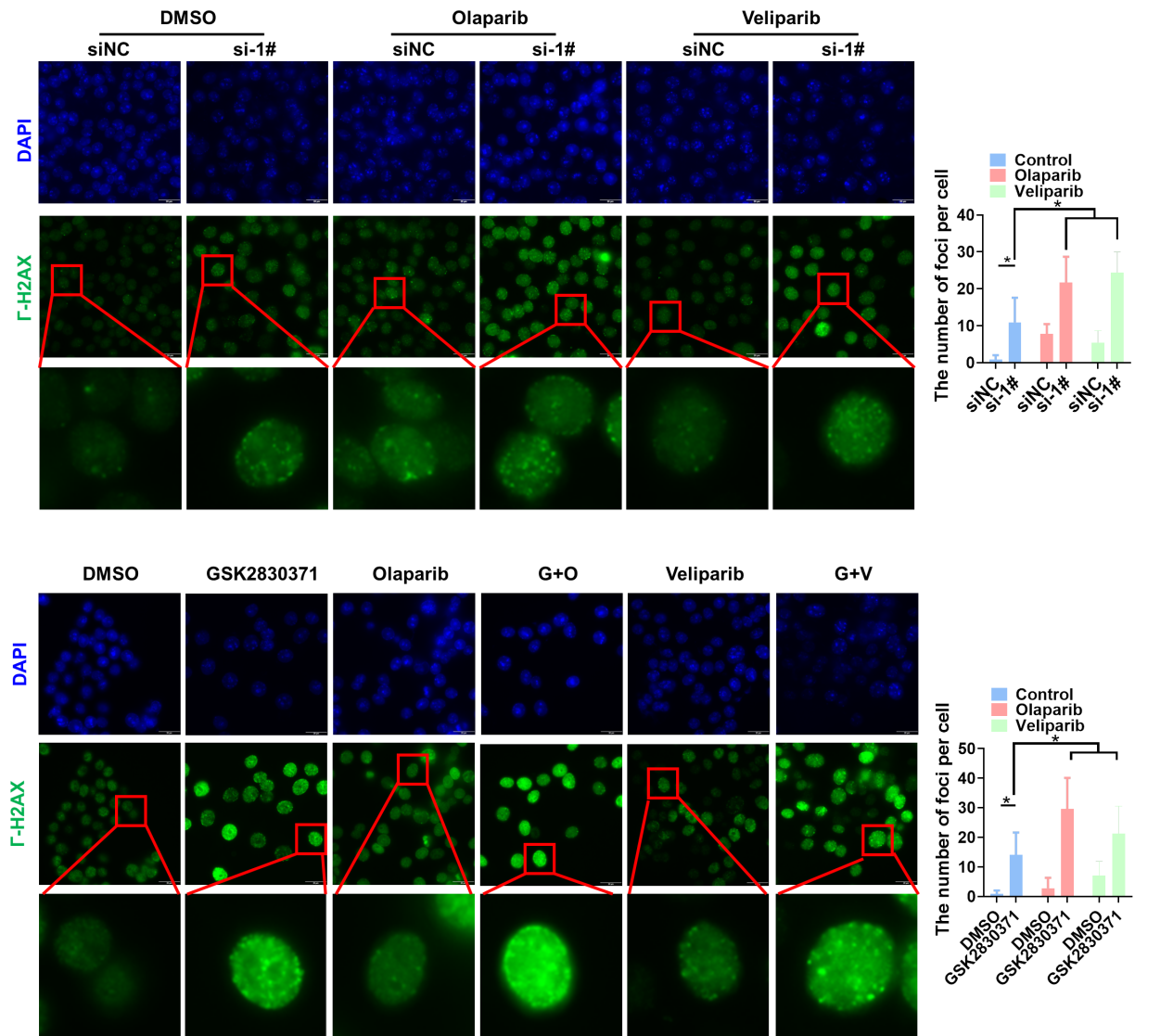


**Supplemental Figure 5. WIP1 and PARP inhibition enhances DNA damage**

A. The foci of γH2AX was measured via immunofluorescence in Hepa1-6 cells after WIP1 knockdown combined with Olaparib (50μM) or Veliparib (50μM) treatment for 24 hours. The numbers of foci were counted, and the average foci number per cell was shown as mean ± SD.

B. The foci of γH2AX was measured via immunofluorescence in Hepa1-6 cells after GSK2830371(25μM) combined with Olaparib (50μM) or Veliparib (50μM) treatment for 24 hours. The numbers of foci were counted, and the average foci number per cell was shown as mean ± SD.


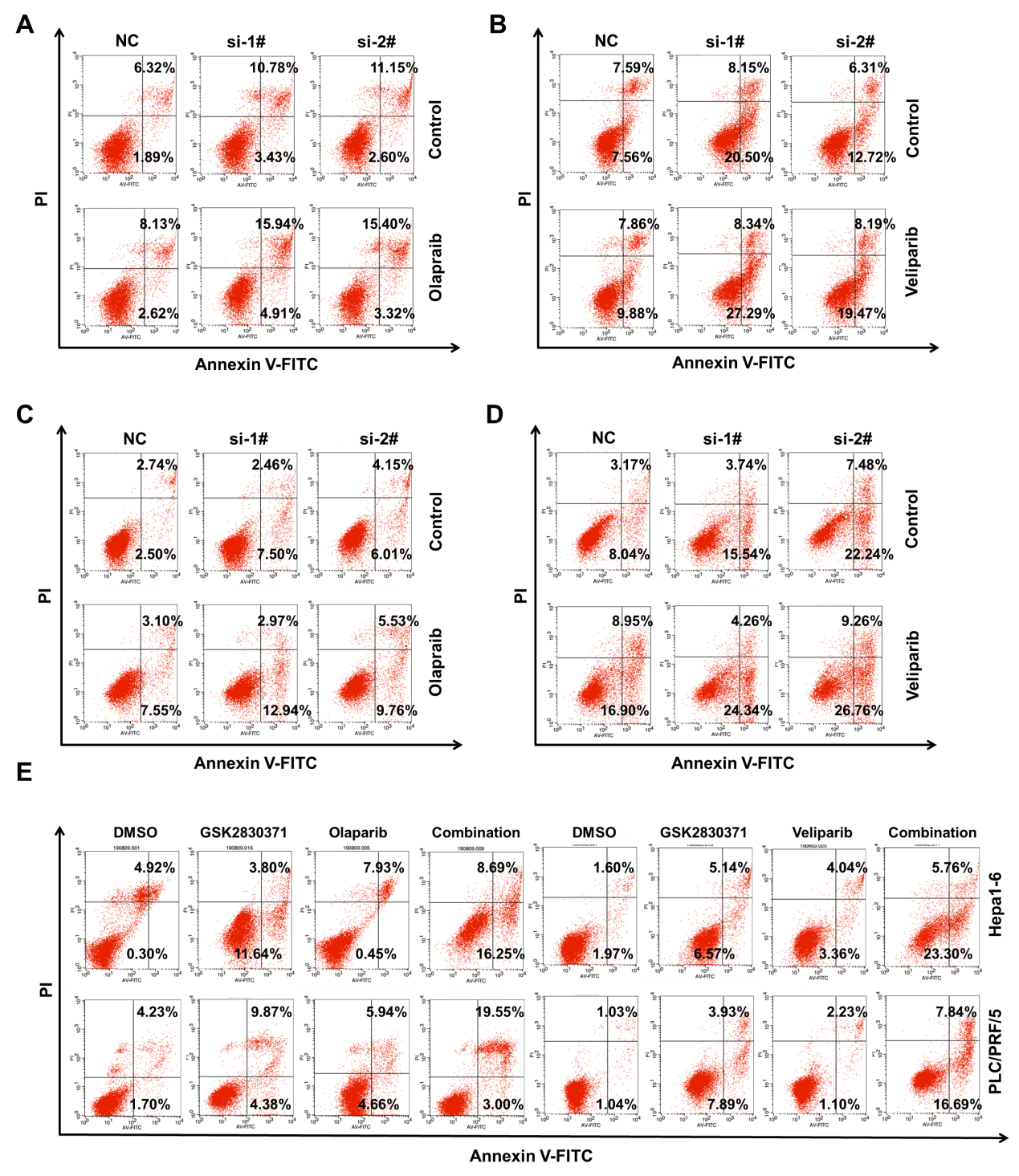


**Supplemental Figure 6. WIP1 and PARP inhibition induce synthetic lethality of HCC cells *in* *vitro***

A-B. The representative apoptosis pictures of Hepa1-6 cells after Olaparib or Veliparib treatments together with WIP1 knockdown measured via flow cytometry with PI and annexin V–FITC double staining.

C-D. The representative apoptosis pictures of PLC/PRF/5 cells after Olaparib or Veliparib treatments together with WIP1 knockdown measured via flow cytometry with PI and annexin V–FITC double staining.

E. The representative apoptosis pictures of Hepa1-6 or PLC/PRF/5 cells after PARPi and/or with GSK2830371 treatments measured via flow cytometry.

**Table S1. siRNA sequences used for knockdown**

| **Name** | **Sequence（5’-3’）** | **Supplier** |
| --- | --- | --- |
| si-PPM1D-homo-1 | GUGCCUACUAAUUCAACAATT  UUGUUGAAUUAGUAGGCACTT | Gene Pharma Company (Shanghai, China) |
| si-PPM1D-homo-2 | GUGGGAGUGUAAUGAACAATT  UUGUUCAUUACACUCCCACTT | Gene Pharma Company (Shanghai, China) |
| si-PPM1D-mus-1 | GCGGCAGUGUGAUGAACAATT  UUGUUCAUCACACUGCCGCTT | Gene Pharma Company (Shanghai, China) |
| si-PPM1D-mus-2 | GGAAUUCAGGAUGACCCAATT  UUGGGUCAUCCUGAAUUCCTT | Gene Pharma Company (Shanghai, China) |

**Table S2. shRNA sequences used for knockdown**

| **Name** | **Sequence（5’-3’）** |
| --- | --- |
| sh-PPM1D-homo-1 | CCCTTCTCGTGTTTGCTTAAACTCGAGTTTAAGCAAACACGAGAAGGG  CCCTTCTCGTGTTTGCTTAAACTCGAGTTTAAGCAAACACGAGAAGGG |
| sh-PPM1D-homo-2 | TGATTTGTGGAGCTATGATTTCTCGAGAAATCATAGCTCCACAAATCA  TGATTTGTGGAGCTATGATTTCTCGAGAAATCATAGCTCCACAAATCA |
| sh-PPM1D-mus-1 | CCTGACTGATAGCCCTACTTACTCGAGTAAGTAGGGCTATCAGTCAGG  CCTGACTGATAGCCCTACTTACTCGAGTAAGTAGGGCTATCAGTCAGG |
| sh-PPM1D-mus-2 | GCCCTGACTTTAAGGATTCATCTCGAGATGAATCCTTAAAGTCAGGGC  GCCCTGACTTTAAGGATTCATCTCGAGATGAATCCTTAAAGTCAGGGC |
